# Chemotherapeutics-induced Oct4 expression contributes to drug resistance and tumor recurrence in bladder cancer

**DOI:** 10.18632/oncotarget.9602

**Published:** 2016-05-26

**Authors:** Chia-Sing Lu, Gia-Shing Shieh, Chung-Teng Wang, Bing-Hua Su, Yu-Chu Su, Yi-Cheng Chen, Wu-Chou Su, Pensee Wu, Wen-Horng Yang, Ai-Li Shiau, Chao-Liang Wu

**Affiliations:** ^1^ Institute of Basic Medical Sciences, College of Medicine, National Cheng Kung University, Tainan, Taiwan; ^2^ Department of Urology, Tainan Hospital, Ministry of Health and Welfare, Executive Yuan, Tainan, Taiwan; ^3^ Department of Biochemistry and Molecular Biology, College of Medicine, National Cheng Kung University, Tainan, Taiwan; ^4^ Department of Internal Medicine, College of Medicine, National Cheng Kung University, Tainan, Taiwan; ^5^ Institute for Science & Technology in Medicine, Keele University, Keele, United Kingdom; ^6^ Department of Urology, College of Medicine, National Cheng Kung University, Tainan, Taiwan; ^7^ Department of Microbiology and Immunology, College of Medicine, National Cheng Kung University, Tainan, Taiwan

**Keywords:** Oct4, drug resistance, tumor recurrence, bladder cancer, all-trans retinoic acid

## Abstract

Cancer cells initially characterized as sensitive to chemotherapy may acquire resistance to chemotherapy and lead to tumor recurrence through the expansion of drug-resistant population. Acquisition of drug resistance to conventional chemotherapy is a major obstacle in the treatment of recurrent cancer. Here we investigated whether anticancer drugs induced Oct4 expression, thereby contributing to acquired drug resistance and tumor recurrence in bladder cancer. We identified a positive correlation of Oct4 expression with tumor recurrence in 122 clinical specimens of superficial high-grade (stages T1-2) bladder transitional cell carcinoma (TCC). Increased Oct4 levels in bladder tumors were associated with short recurrence-free intervals in the patients. Chemotherapy induced Oct4 expression in bladder cancer cells. Notably, treatment with cisplatin increased CD44-positive bladder cancer cells expressing Oct4, representing cancer stem-like cell subpopulation. Forced expression of Oct4 reduced, whereas knockdown of Oct4 enhanced, drug sensitivity in bladder cancer cells. Furthermore, tumor cells overexpressing Oct4 responded poorly to cisplatin *in vivo*. In regard to clinical relevance, inhibition of Oct4 by all-*trans* retinoic acid (ATRA) synergistically increased sensitivity to cisplatin in bladder cancer cells. Furthermore, the combination of cisplatin and ATRA was superior to cisplatin alone in suppressing tumor growth. Therefore, our results provide evidence that Oct4 increases drug resistance and implicate that inhibition of Oct4 may be a therapeutic strategy to circumvent drug resistance.

## INTRODUCTION

Cancer cells that have been initially characterized as sensitive to chemotherapy may acquire resistance to chemotherapy and lead to tumor recurrence through the expansion of drug-resistant population. Platinum-based drugs such as cisplatin are the standard first-line agents used alone or in combination with other drugs for cancer treatment. However, tumors can recur and develop chemoresistance [1]. Solid tumors possess a rare population of cancer stem cells (CSCs), which have similar characteristics to normal stem cells and exhibit self-renewal, asymmetric cell division, and resistance to toxic agents [2]. CSCs display surface markers, including CD44 and CD133, in various cancer types [3]. CD44 and CD133 have been identified as surface biomarkers for cancer cells resistant to chemotherapeutic drugs [4–6]. CSC-like cells are enriched following short-term single treatment of chemotherapy, suggesting that malignant cells may also be enriched by cisplatin treatment [6].

Oct4, a member of POU homeobox gene family, is a transcription factor capable of binding to an octameric consensus sequence to activate its target genes [7]. It functions in maintaining pluripotency and self-renewal of embryonic stem (ES) cells [8]. Oct4 is highly expressed in lung cancer-derived CD133- and CD44-positive cells, anticancer drug-selected breast cancer cells, and side population (SP) cells from bladder and ovarian cancer cells with characteristics of CSCs [9–13], which exhibit enhanced resistance to chemotherapeutic agents, such as cisplatin. We have previously shown that Oct4 is detected and predicts tumor progression and metastasis in bladder cancer [14]. Moreover, we have also demonstrated that Oct4-regulated oncolytic adenovirus can kill CD44- and CD133-positive bladder cancer cells [15]. Expression of Oct4 in CSC-like cell population not only promotes cell growth, but also resists chemotherapy [16], suggesting a role for Oct4 in regulating drug resistance.

Bladder carcinoma is the most common urothelial malignancy in more developed countries [17]. Transitional cell carcinoma (TCC) is the most common bladder tumor and ~90% of bladder TCC are superficial at initial diagnosis. Although primary tumors can be eliminated by surgery, chemotherapy, or radiotherapy, the tumors recur frequently and may progress to muscle-invasive disease [18]. The acquisition of drug resistance in recurrent tumors is a critical factor that limits successful cancer treatment. Resistance to common chemotherapeutic agents involves multiple mechanisms, which can be intrinsic or acquired during treatment [19]. Since acquired drug resistance is an important contributor to the recurrence and progression of bladder cancer, elucidating its underlying mechanisms may provide novel therapeutic strategies for bladder cancer.

In the present study, we investigated whether anticancer drugs induced Oct4 expression, thereby contributing to acquired drug resistance and tumor recurrence in bladder cancer. Our results show that expression of Oct4 was positively correlated with tumor recurrence in clinical specimens of bladder cancer. Our *in vitro* and *in vivo* studies also demonstrate that induction of Oct4 expression after treatment with anticancer drugs rendered bladder cancer cells chemoresistant. In conclusion, our results provide evidence that Oct4 can induce drug-acquired chemoresistance in bladder cancer, and implicate that inhibition of Oct4 may be further explored as a therapeutic strategy to counteract acquired drug resistance.

## RESULTS

### Expression levels of Oct4 are increased in recurrent bladder cancer and positively correlated with tumor recurrence

**Figure 1:**
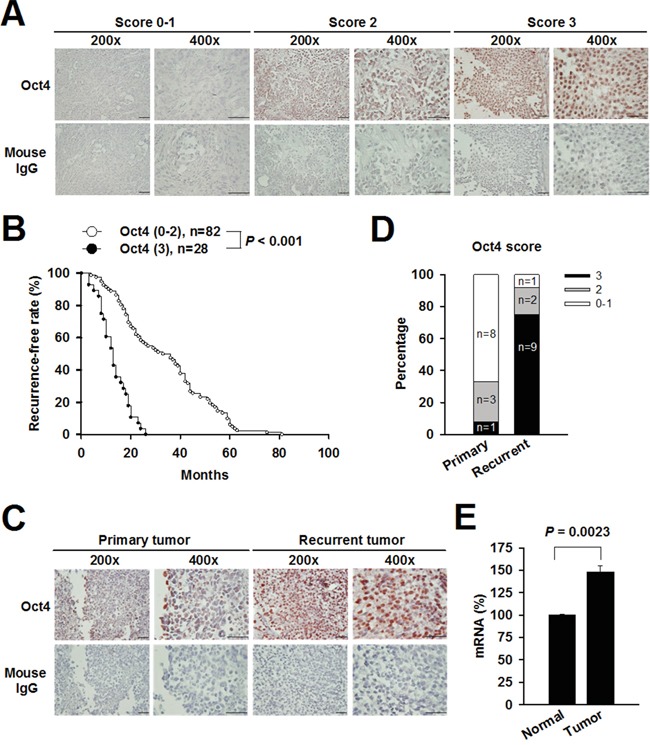
High levels of Oct4 expression are correlated with tumor recurrence in patients with high-grade superficial bladder TCC **A**. Immunohistochemical detection of Oct4 expression in three representative tumor sections (scores 0-1, 2, and 3). The immunoreactivity was scored on the basis of the percentage of positive cells (positive staining in <30% (scores 0-1), 30%-70% (score 2), and >70% (score 3) of cells). Negative control slides stained with isotype control mouse IgG were included. **B**. Kaplan-Meier curves of recurrence-free rate in 110 patients with high (score 3) or low (score 0-2) Oct4 expression. Differences in recurrence-free intervals were analyzed by the log-rank test. **C**. Representative immunohistochemical staining of Oct4 in paired primary and recurrent tumor specimens. Negative control slides stained with isotype control mouse IgG were included. **D**. Percentages of primary (n = 12) and recurrent (n = 12) tumors that expressed Oct4 with different scores (*P* < 0.001, Mann-Whitney U test). **E**. Relative mRNA levels of Oct4 in human bladder tumor and normal tissue determined by real-time RT-PCR analysis. Values shown are the mean ± SEM (n = 3). Results are representatives of two independent experiments.

As expression of Oct4 is associated with tumor progression and chemotherapy resistance, we first examined the expression levels of Oct4 in 122 clinical specimens of superficial high-grade (stages T1-2) bladder TCC of 110 patients, among which 24 specimens were paired primary and recurrent samples from 12 patients. Figure 1A shows that the immunoreactive intensity for Oct4 was individually scored as 0-3 and categorized as low (0-1), moderate (2), or high (3) [14, 20]. Kaplan-Meier analysis reveals that patients with Oct4 high-expressing tumors (score 3) had significantly shorter recurrence-free intervals (median = 13 months) than those with Oct4 low-expressing tumors (score 0-2) (median = 34.5 months) (*P* < 0.001) (Figure 1B). We further analyzed the expression levels of Oct4 in 12 paired primary and recurrent tumor specimens. Expression levels of Oct4 in recurrent tumors were higher than those in their primary tumor counterparts (Figure 1C). As shown in Figure 1D, much higher percentages of tumors with high expression (score 3) of Oct4 and much lower percentages of tumors with low expression (score 0-1) of Oct4 were noted in recurrent tumors than in primary tumors (*P* < 0.001). We also used quantitative real-time reverse transcription-polymerase chain reaction (RT-PCR) analysis to assess Oct4 expression in bladder tumor and normal tissue. Figure 1E show that mRNA levels of Oct4 were significantly higher in bladder tumor than in normal tissue. Taken together, we identified a positive correlation of Oct4 expression with bladder tumor recurrence.

### Expression of Oct4 is increased in bladder cancer cells following chemotherapeutic treatment

**Figure 2:**
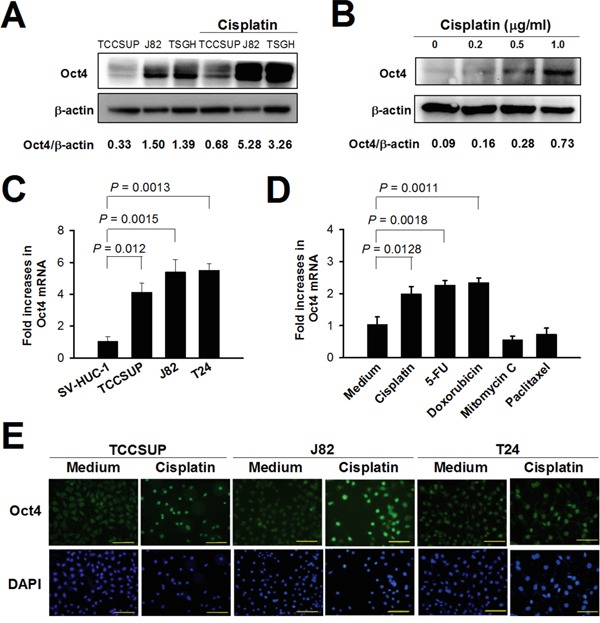
Treatment with cisplatin increases Oct4 expression in bladder cancer cells **A, B**. Detection of Oct4 in TCCSUP, J82, and TSGH-8301 cells treated with cisplatin (1 μg/ml) (A) and in TCCSUP cells treated with various concentrations of cisplatin (B) for 24 h by immunoblotting. Expression of β-actin serves as the loading control. Ratios between the intensity of the bands corresponding to Oct4 and those corresponding to β-actin, which were quantitated by densitometry, were calculated. **C, D**. Detection of mRNA levels of Oct4 in bladder cancer cells (TCCSUP, J82, and T24 cells) and immortalized uroepithelial SV-HUC-1 cells (C) and in TCCSUP cells after treatment with various chemotherapeutic drugs at IC_50_ concentrations for 72 h (D) by quantitative real-time RT-PCR analysis. Values shown are the mean ± SEM (n = 3). Results are representatives of two independent experiments. **E**. Detection of Oct4 in TCCSUP, J82, and T24 cells following cisplatin treatment by double immunofluorescence staining. The cells were treated with cisplatin (1 μg/ml) or left untreated for 48 h. They were stained with mouse anti-Oct4 monoclonal antibody, and subsequently stained with Alexa Fluor488-goat anti-mouse IgG. The nucleus was counterstained with DAPI. Expression and localization of Oct4 were observed under fluorescence microscopy at a magnification of ×400. Scale bar, 50 μm.

We investigated whether Oct4 was involved in acquired resistance induced by anticancer drugs. As shown in Figure 2A, protein levels of Oct4 were dramatically increased in TCCSUP, J82, and TSGH-8301 bladder cancer cells after treatment with 1 μg/ml of cisplatin for 24 h. Figure 2B shows that TCCSUP cells expressed Oct4 upon cisplatin treatment in a dose-dependent manner. Bladder cancer cells, including TCCSUP, J82, and T24 cells, expressed higher levels of Oct4 mRNA than SV-HUC-1 normal human urothelial cells, as determined by real-time quantitative RT-PCR analysis (Figure 2C). The mRNA levels of Oct4 were increased in TCCSUP cells treated with cisplatin, 5-fluorouracil (5-FU), and doxorubicin, whereas treatment with mitomycin C or paclitaxel (Taxol) had no such effects (Figure 2D). Furthermore, Oct4 protein induced by cisplatin in different bladder cancer cells was located in the nucleus (Figure 2E). Collectively, these results suggest that expression of Oct4 was enhanced in bladder cancer cells that had been exposed to various anticancer drugs.

### CD44-positive cells expressing Oct4 are increased in bladder cancer cells following cisplatin treatment

**Figure 3:**
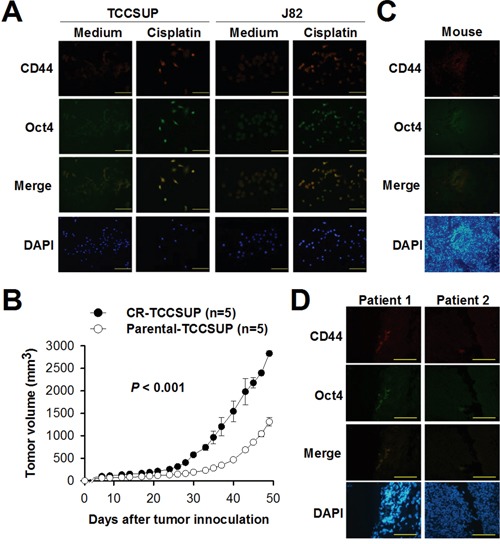
Treatment with cisplatin increases CD44-positive cells expressing Oct4 in bladder cancer **A**. Detection of CD44 and its colocalization with Oct4 in TCCSUP and J82 cells by double immunofluorescence staining. Cells were treated with cisplatin (1 μg/ml) or left untreated for 24 h. They were stained with PE-conjugated mouse anti-CD44 monoclonal antibody and mouse anti-Oct4 monoclonal antibodies, and subsequently stained with Alexa Fluor488-goat anti-mouse IgG. Expression and colocalization of CD44 and Oct4 were observed under fluorescence microscopy at a magnification of ×200. Scale bar, 100 μm. The merged column represents the superposition of the cells stained with anti-CD44 and anti-Oct4. **B**. Tumor volumes in mice bearing parental or cisplatin-resistant TCCSUP tumors after cisplatin treatment. NOD/SCID mice were inoculated subcutaneously with parental TCCSUP or cisplatin-resistant TCCSUP (CR-TCCSUP) cells (1 × 10^7^) at day 0, followed by intraperitoneal injection of cisplatin (4 mg/kg) at days 5, 7, 9, 11 and 13. Values shown are the mean ± SEM (n = 5). **C, D**. Detection of CD44 and its colocalization with Oct4 in human bladder tumor xenografts obtained from the tumor-bearing mice (C) and in human bladder tumor tissue of cancer patients (D). Tumors were excised at day 50 from the mice that had been inoculated with CR-TCCSUP cells and treated with cisplatin as described in B. Human tumor specimens were obtained from two bladder cancer patients. Double immunofluorescence staining was performed as described in A. The nucleus was counterstained with DAPI. Scale bar, 100 μm.

Since Oct4 is considered a key maintainer of CSC pluripotency, we next explored whether expression of Oct4 and CD44 was increased in bladder cancer cells following cisplatin treatment. Immunofluorescence analysis show that treatment with cisplatin enhanced the expression of both CD44 and Oct4 in bladder cancer cells (Figure 3A), Notably, CD44 was colocalized with Oct4, suggesting concurrent upregulation of CD44 and Oct4 during cisplatin treatment. Moreover, we compared the tumor growth of NOD/SCID mice inoculated with parental TCCSUP cells and that inoculated with cisplatin-resistant TCCSUP cells following 5 doses of cisplatin treatment every other day from day 5 after tumor cell inoculation. Figure 3B shows that cisplatin-resistant TCCSUP tumors grew faster and larger than the parental tumors (*P* < 0.001), indicating that drug-resistant tumors progressed more rapidly than did parental tumors, which may be attributed to the elevation of Oct4 after chemotherapy. Expression of Oct4 in the CD44-positive tumor cells could also be detected, albeit in a small percentage, in the tissue slices of cisplatin-resistant TCCSUP tumor xenografts after cisplatin treatment (Figure 3C), suggesting a potential role for Oct4 in the poor response of tumors to cisplatin treatment. Colocalization between Oct4 and CD44 expression was also observed in the clinical tumor specimens from two patients with bladder cancer (Figure 3D). Taken together, these results suggest that CD44-positive cancer cells expressing Oct4 are induced by cisplatin, which may contribute to drug resistance.

### Overexpression of Oct4 in bladder cancer confers resistance to cisplatin *in vitro* and *in vivo*

**Figure 4:**
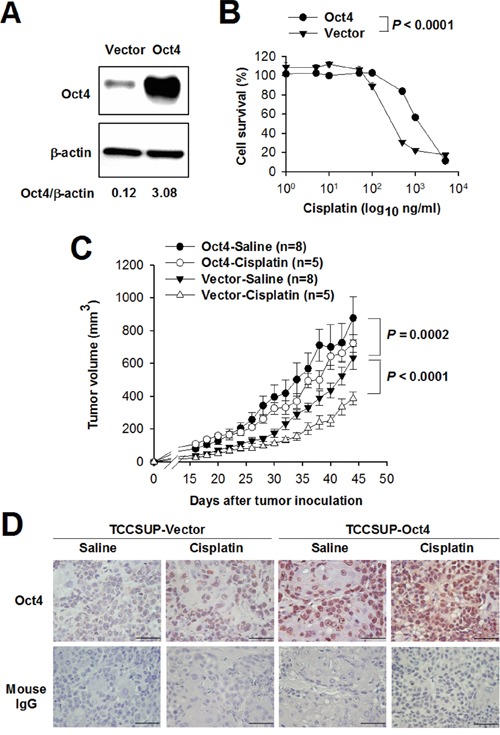
Overexpression of Oct4 confers resistance to cisplatin in bladder cancer **A**. Detection of Oct4 in TCCSUP cells that had been transduced with lentiviral vectors expressing Oct4 or with control lentiviral vectors by immunoblotting. Expression of β-actin serves as the loading control. Ratios between the intensity of the bands corresponding to Oct4 and those corresponding to β-actin, which were quantitated by densitometry, were calculated. **B**. A five-fold increase in the IC_50_ value of cisplatin in Oct4-overexpressing cancer cells. Oct4-overexpressing TCCSUP or control cells were treated with various concentrations of cisplatin for 72 h. Cell viability was assessed by the WST-8 assay to determine the IC_50_ value. Values represent the relative cell survival, with the viability in the control cells without cisplatin treatment arbitrarily set to 100. Values shown are the mean ± SEM (n = 4). **C**. Tumor volumes in mice bearing Oct4-overexpressing or control TCCSUP tumors treated with cisplatin or saline. NOD/SCID mice were inoculated subcutaneously with Oct4-overexpressing or control TCCSUP cells (2 × 10^6^) at day 0, followed by intraperitoneal injection of cisplatin (4 mg/kg) or saline when tumor volumes reached 100 mm^3^. Values shown are the mean ± SEM (n = 5-8). **D**. Immunohistochemical staining for Oct4 in the tumors excised at day 45 from the mice described in C (×400 magnification, scale bar, 50 μm). Negative control slides stained with isotype control mouse IgG were included.

We next investigated whether Oct4 contributed to cisplatin-induced acquired drug resistance. We generated Oct4-overexpressing and vector control TCCSUP cells using lentivirus-mediated gene transfer (Figure 4A). Overexpression of Oct4 resulted in a five-fold increase in the 50% inhibitory concentration (IC_50_) value of cisplatin in Oct4-overexpressing TCCSUP cells (3.43 ± 0.2 μM) compared to their control counterparts (0.67 ± 0.086 μM) (*P* < 0.0001) (Figure 4B). To verify the contribution of Oct4 to drug resistance *in vivo*, we treated NOD/SCID mice bearing human TCCSUP-vector or TCCSUP-Oct4 xenografts with cisplatin or saline. As shown in Figure 4C, mice bearing TCCSUP-Oct4 tumors had larger tumor volumes than those bearing TCCSUP-vector tumors without cisplatin treatment (*P* = 0.0002). Whereas treatment with cisplatin significantly retarded tumor growth in mice bearing TCCSUP-vector tumors (*P* < 0.0001), it had no effects on reducing tumor growth in mice bearing TCCSUP-Oct4 tumors compared to treatment with saline. These results indicate that Oct4-overexpressing tumors responded poorly to cisplatin treatment. At day 44 after tumor cell inoculation, tumors were resected from the tumor-bearing mice for immunohistochemical examination. As shown in Figure 4D, TCCSUP-vector tumors, in particular TCCSUP-Oct4 tumors, expressed higher levels of Oct4 following cisplatin treatment compared with those without treatment. Collectively, overexpression of Oct4 in cisplatin-treated mice suggests that the mechanism underlying acquired drug resistance may involve Oct4 overexpression.

### Knockdown of Oct4 expression enhances sensitivity of bladder cancer cells to various chemotherapeutic agents

To further verify the correlation of Oct4 expression with drug resistance in bladder cancer cells, we generated lentivirus-mediated stable Oct4-knockdown cells and their control cells. In the Oct4-knockdown TCCSUP, expression of Oct4 transcripts was suppressed to various degrees in two different knockdown cells (Supplementary Figure S1). Susceptibilities of Oct4-knockdown cells to various chemotherapeutic agents were examined. Table 1 shows that except for mitomycin C, knockdown of Oct4 expression rendered TCCSUP cells more susceptible to the chemotherapeutic agents tested, including cisplatin, 5-FU, doxorubicin, paclitaxel, gemcitabine, and methotrexate. Taken together, these results demonstrate that suppression of Oct4 expression in bladder cancer cells results in increased sensitivity to various chemotherapeutic agents.

**Table 1:** IC_50_ values of various chemotherapeutic agents in Oct4-knockdown and control TCCSUP cells

Drug	shLuc	shOct4				
		TRCN4882		TRCN4883		
	IC_50_	IC_50_	*P*	IC_50_	*P*	Unit
Cisplatin	0.85 ± 0.12	0.44 ± 0.09	***	0.70 ± 0.21	*	μM
5-FU	0.97 ± 0.27	0.38 ± 0.01	***	0.46 ± 0.09	**	μM
Doxorubicin	11.9 ± 2.59	6.21 ± 0.86	***	10.17 ± 2.41	***	nM
Mitomycin C	93.02 ± 11.96	93.32 ± 19.44	N.S.	86.14 ± 17.95	N.S.	nM
Paclitaxel	5.74 ± 0.47	2.69 ± 0.23	***	3.51 ± 0.35	***	nM
Gemcitabine	37.71 ± 1.00	6.01 ± 1.00	***	11.35 ± 0.67	***	nM
Methotrexate	17.38 ± 1.32	8.58 ± 0.44	***	13.64 ± 0.66	***	nM

TCCSUP cells (2 × 10^3^) that had been transduced with lentiviral vectors expressing shOct4 or shLuc were seeded in 96-well plates overnight and refed with fresh medium containing different concentrations of chemotherapeutic agents. After 72 h, cell viability was assessed with the WST-8 assay to determine IC_50_ values. Values shown are the mean ± SD from three independent experiments. Statistical differences in the IC_50_ value between shOct4 and shLuc knockdown cells were analyzed by two-way ANOVA with Bonferroni post hoc test. *, *P* < 0.05; **, *P* < 0.01; ***, *P* < 0.001; N.S., non-significant.

### All-*trans* retinoic acid (ATRA) synergistically enhances cisplatin-induced cytotoxicity in bladder cancer cells

**Figure 5:**
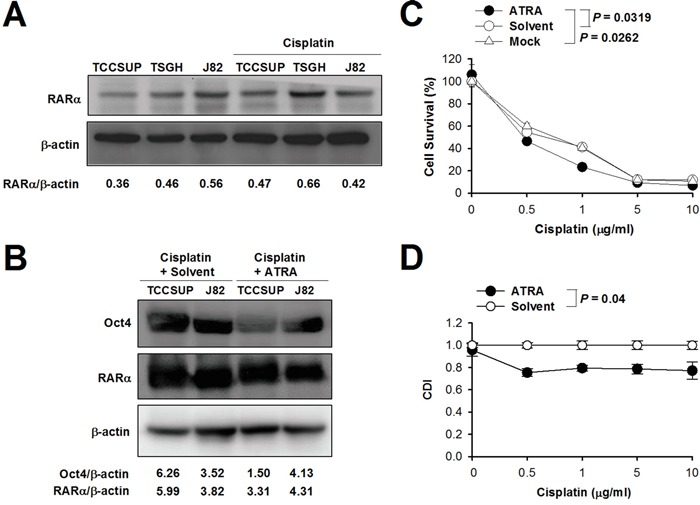
Combination treatment with cisplatin and ATRA increases sensitivity to cisplatin in bladder cancer cells **A, B**. Detection of RARα in TCCSUP, TSGH-8301, and J82 cells treated with or without cisplatin (1 μg/ml) for 24 h (A), and Oct4 and RARα in TCCSUP and J82 cells treated with cisplatin (1 μg/ml) in the presence of ATRA (2.5 μM) or the vehicle (solvent) for 24 h (B) by immunoblotting. Expression of β-actin serves as the loading control. Ratios between the intensity of the bands of the indicted proteins and those corresponding to β-actin, which were quantitated by densitometry, were calculated. **C**. Cell viability after treatment with cisplatin alone or combined with ATRA. TCCSUP cells were treated with various concentrations of cisplatin plus ATRA (2.5 μM), saline, or the vehicle (solvent). After 72 h, cell viability was determined with the WST-8 assay to determine the IC_50_ values. **D**. Synergistic effect of cisplatin plus ATRA. TCCSUP cells were treated as described in C, except that 0.1 μM of ATRA was used. The values of the CDI for the combination treatment of indicated concentrations of cisplatin plus ATRA were calculated.

ATRA, a powerful differentiating agent, can suppress Oct4 gene expression because it can influence multiple signaling pathways involved in stem cell maintenance [21]. ATRA is transported into the nucleus by binding to retinoid acid receptor α (RARα). Expression of RARα was detected in TCCSUP, TSGH-8301, and J82 cells, and no significant changes were found after treatment with cisplatin (Figure 5A). We further investigated the effect of ATRA on the expression of Oct4 and RARα in cisplatin-treated TCCSUP and J82 cells. As shown in Figure 5B, treatment with ATRA significantly decreased the expression of Oct4 and RARα in TCCSUP cells in the presence of cisplatin. However, such effects were not evident in J82 cells. To further determine whether treatment with ATRA in bladder cancer cells could increase their sensitivity to cisplatin, we combined ATRA with cisplatin to treat bladder cancer cells. The IC_50_ value of cisplatin in TCCSUP cells was significantly lower in ATRA-treated cells than in the vehicle (solvent)-treated control cells (mean ± SEM, 1.31 ± 0.19 *versus* 1.91 ± 0.27 μM; *P* = 0.0319) (Figure 5C). We next evaluated the combination effect of cisplatin and ATRA on the cytotoxicity of TCCSUP cells using the coefficient of drug interaction (CDI) [22–24]. The value of the CDI for the combination of cisplatin (ranging from 0.5 to 10 μg/ml) with ATRA (0.1 μM) was less than 1, indicative of a synergistic effect (Figure 5D).

### Combination treatment of cisplatin with ATRA inhibits the growth of bladder tumor

**Figure 6:**
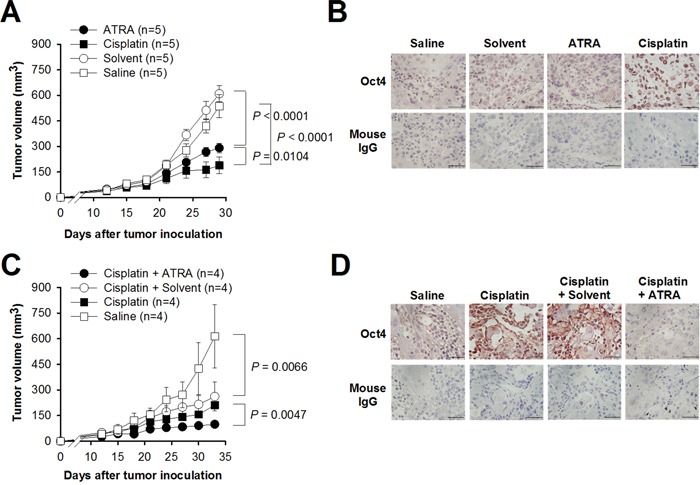
Combination treatment with cisplatin and ATRA is superior to cisplatin alone in suppressing bladder tumor growth *in vivo* **A**. Tumor volumes in TCCSUP tumor-bearing mice receiving cisplatin or ATRA alone. TCCSUP cells (2 × 10^6^) were subcutaneously inoculated into NOD/SCID mice. When tumor volumes reached 100 mm^3^, the mice were treated with cisplatin (4 mg/kg), ATRA (10 mg/kg), saline, or the solvent alone. **B**. Immunohistochemical staining for Oct4 in the tumors excised at day 30 from the mice described in A (×400 magnification, scale bar, 50 μm). Negative control slides stained with isotype control mouse IgG were included. **C**. Tumor volumes in TCCSUP tumor-bearing mice receiving cisplatin alone or in combination with ATRA. Tumor-bearing mice as described in A were treated with cisplatin (4 mg/kg) with or without ATRA (10 mg/kg). **D**. Immunohistochemical staining for Oct4 in the tumors excised at day 34 from the mice described in C (×400 magnification, scale bar, 50 μm). Negative control slides stained with isotype control mouse IgG were included.

We next evaluated the effects of cisplatin and ATRA alone or in combination on the growth of bladder tumor *in vivo*. We treated TCCSUP tumor-bearing mice with ATRA, cisplatin, the vehicle (solvent), or saline alone and monitored their tumor growth. Single treatment with either cisplatin or ATRA significantly slowed tumor growth compared to their control counterparts, with cisplatin being more effective than ATRA (Figure 6A). Nevertheless, mice treated with cisplatin expressed higher levels of Oct4 compared with the remaining three groups of mice (Figure 6B). As shown in Figure 6C, although treatment with cisplatin plus solvent significantly retarded tumor growth compared with saline treatment in TCCSUP tumor-bearing mice (*P* = 0.0066), combination treatment with cisplatin and ATRA was superior to single treatment with cisplatin (*P* = 0.0047). Compared with saline-treated group, higher levels of Oct4 in the tumors were detected in mice receiving cisplatin alone or in combination with the vehicle (solvent) (Figure 6D). By contrast, combination treatment with cisplatin and ATRA abrogated cisplatin-induced Oct4 expression. Therefore, these results suggest that induction of Oct4 may be involved in the acquired drug resistance induced by cisplatin. Furthermore, ATRA, which inhibits Oct4 expression, may provide potential therapeutic benefits toward the effectiveness of chemotherapeutic agents for cancer therapy.

## DISCUSSION

Early detection of cancer has been improved in recent years; however, the prognosis of patients with some cancers especially those with late stage remains poor, mostly due to development of drug resistance, followed by tumor recurrence. In the present study, we analyzed 122 clinical specimens of superficial high-grade bladder TCC of 110 patients for Oct4 expression by immunohistochemistry. Patients with high expression levels of Oct4 were associated with short recurrence-free intervals. Expression levels of Oct4 in recurrent tumors were significantly higher than those in primary tumors. We further investigated a potential role for Oct4 in drug resistance of bladder cancer *in vitro* and in mice. Treatment with various chemotherapeutic drugs increased Oct4 expression in bladder cancer cells. Moreover, overexpression of Oct4 reduced, whereas knockdown of Oct4 enhanced, sensitivity to anticancer drugs. Collectively, our results indicate that Oct4 increases the resistance of bladder cancer against various chemotherapeutic agents. However, the exact role of Oct4 overexpression in drug resistance and its underlying molecular mechanism have yet to be elucidated.

The unique capabilities of proliferation, tumorigenesis, and chemoresistance of CSCs make these cells an attractive therapeutic target and an ideal candidate for developing strategies aiming at preventing cancer recurrence. CD44 and CD133 are the most common CSC markers and broadly expressed on cancer cells [25–28]. Although there are various CSC markers, such as CD133, CD44, and ALDH1, displayed on cancer cells, Oct4 expression in these cells plays a major role in anti-apoptosis and maintenance of pluripotency. In the present study, we show that CD44-positive cells that expressed Oct4 were dramatically increased in bladder cancer cell lines after cisplatin treatment. Moreover, colocalization of CD44 with Oct4 was detectable, albeit in a small percentage of cells, in human bladder tumor xenografts that were resistant to cisplatin treatment. Our results suggest that bladder cancer cells undergo CSC-like changes after exposure to cisplatin. Genetic instability or environmental stimulation may result in genetic or epigenetic changes, revealing the flexibility of cancer cells. The chemotherapy-induced stemness phenotype of cancers is acquired and maintained by Oct4 [29]. During cisplatin treatment, activation of the *Oct4* gene was attributed to decreased promoter methylation [16]. Oct4 can enhance survivin expression to promote cancer cell proliferation [30] and is critical for survival/anti-apoptosis of murine ES cells [31]. Our results show that the induction of Oct4 enhances the acquisition of CSC phenotypes and resistance to cisplatin in bladder cancer cells. In addition to cisplatin, treatment of lung cancer cells with 5-FU, doxorubicin, etoposide, and methotrexate has been shown to enrich CSC-like cells with drug resistance [32].

Transcription factors are involved in the regulation of stress-inducible genes. Oct1, which belongs to the same POU homeobox gene family as Oct4, is induced after cells are exposed to different DNA-damaging agents, such as cisplatin [33]. Furthermore, different transcription factors have been shown to play roles in the acquisition of cellular resistance to cisplatin, such as NF-κB, YB-1, and activating transcription factor 4 [34]. In the current study, we demonstrate that treatment with cisplatin, 5-FU, and doxorubicin, but not mitomycin C and paclitaxel, induces Oct4 expression. Moreover, knockdown of endogenous Oct4 expression sensitizes bladder cancer cells to cisplatin, 5-FU, doxorubicin, paclitaxel, gemcitabine, and methotrexate, but not to mitomycin C. Despite the fact that Oct4 is not induced following paclitaxel treatment, knockdown of Oct4 enhances sensitivity of bladder cancer cells to paclitaxel. Notably, mitomycin C fails to induce Oct4 expression. Furthermore, knockdown of Oct4 expression in bladder cancer cells does not affect their sensitivity to mitomycin C. Taken together, these results suggest that Oct4 either directly or indirectly contributes to the cross-resistance of bladder cancer cells to the chemotherapeutic drugs used for patients with bladder cancer, including cisplatin, 5-FU, doxorubicin, paclitaxel, gemcitabine, and methotrexate. However, resistance mechanism of mitomycin C in bladder cancer is independent of Oct4. Given multiples functions of Oct4 in cancer cells, further studies are required to elucidate the mechanisms underlying the role of Oct4 in the resistance of bladder cancer cells to different anticancer drugs.

Patients with metastatic bladder cancer are treated with cisplatin-containing systemic chemotherapy, such as gemcitabine plus cisplatin, as the standard first-line therapy. However, treatment failure is commonly caused by the development of drug resistance. Resistance mechanisms to cisplatin in cancer cells include reduced cellular uptake, increased efflux, increased DNA repair, and hypermethylation of the *MLH1* gene, a mismatch repair gene [19]. In the present study, we demonstrate that treatment with cisplatin increases expression of Oct4, contributing to chemoresistance and tumor recurrence. Our data can explain, in part, the high recurrence rate and drug resistance of bladder cancer from neoadjuvant chemotherapy. However, large-scale studies on the comparison between neoadjuvant and postoperative chemotherapy should be conducted for further evaluation.

The multidrug resistant (MDR) phenotype has been widely recognized in chemotherapy for bladder cancer [35]. The ATP-binding cassette (ABC) transporter family of transmembrane proteins have been linked to resistance of doxorubicin, paclitaxel, methotrexate, and mitomycin C by promoting drug efflux in various cancer types [36]. For cisplatin resistance, limited intracellular accumulation of cisplatin most often derives from reduced uptake, rather than increased efflux [37]. Mitomycin C, a DNA cross-linking agent, is unique as an anticancer drug in that it is preferentially converted to an active form through enzymatic reduction in hypoxic regions of solid tumors [38]. It is thus more toxic to hypoxic cells than to aerobic cells [39, 40]. Regarding antimetabolites 5-FU, gemcitabine, and methotrexate used in the present study, increased expression of thymidylate synthase, which is the target of 5-FU, was shown to be a resistance mechanism to 5-FU [41]. Dysregulation of the enzymes participating in the gemcitabine metabolic pathway is one of the mechanisms responsible for gemcitabine resistance [42]. Amplification of the dihydrofolate reductase (DHFR) gene resulting in increased levels of the enzyme was identified as one mechanism of acquired methotrexate resistance [43]. Given the complexity and heterogeneity of bladder cancer as well as the variety of mechanisms involved in drug resistance, further studies are warranted to uncover the molecular basis by which Oct4 induces acquired resistance of bladder cancer to different anticancer drugs.

In the management of cancer patients, drug resistance is a major cause of treatment failure. Our results indicate that suppression of Oct4 expression may improve drug susceptibility to overcome the acquired resistance to chemotherapeutic drugs. ATRA, a derivative of vitamin A, can promote ES cell differentiation by repressing Oct4 gene expression through several retinoic acid-responsive elements (RAREs) located in the promoter-enhancer region of the *Oct4* gene [21]. We have previously shown that inhibition of Oct4 expression by ATRA in bladder cancer cells renders cells less susceptible to cytolytic effects induced by an oncolytic adenovirus carrying the Oct4 response element (ORE) [44]. In the present study, we demonstrate that inhibition of Oct4 expression by ATRA improves the sensitivity of cancer cells to cisplatin. ATRA is used as an anticancer agent for acute promyelocytic leukemia [45]. The combined treatment with ketoconazole and ATRA for bladder cancer after transurethral resection of bladder tumor (TURBT) significantly improved the survival and reduced recurrence through decreasing levels of vascular endothelial growth factor (VEGF) and transforming growth factor-α (TGF-β) [46]. In addition, when compared with methotrexate, vinblastine, and doxorubicin plus cisplatin, combination treatment with gemcitabine plus cisplatin offered a similar survival result but with a better safety profile and tolerability in patients with bladder cancer [47, 48]. In the present study, we demonstrate the synergistic cytotoxicity of ATRA combined with cisplatin, suggesting that inhibition of Oct4 may be a potentially effective therapeutic strategy against cancer and provide an additional benefit for chemotherapy and/or radiotherapy.

ATRA exerts pleiotropic effects on cell proliferation and differentiation, which can be independent of its effect on Oct4 gene expression [45]. It can promote the differentiation of dendritic cells in cancer patients [49]. It has also been suggested to modulate the MAPK pathway [50] and repress invasion and stem cell phenotypes by induction of metastasis suppressors through RAREs [51]. Therefore, ATRA provides multiple advantages for cancer therapy because it induces differentiation of CSC-like cells, rendering them sensitive to chemotherapy. ATRA-mediated Oct4 inhibition may not only suppress the promoter activity of Oct4, but also be through the degradation of Pin1, a peptidyl-prolyl isomerase. Pin1 is induced upon cellular reprogramming and enhances generation of induced pluripotent stem (iPS) cells [52]. Pin1 interacts with the phosphorylated Ser12-Pro motif of Oct4, which in turn facilitates the stability and transcriptional activity functions of Oct4. Thus, ATRA simultaneously blocks multiple Pin1-regulated cancer-driving pathways, an attractive property for treating aggressive and drug-resistant tumors [53].

In the present study, we have provided evidence that Oct4 expression is enhanced in bladder cancer cells after treatment with various chemotherapeutic agents, rendering bladder cancer chemoresistant. In a broad sense, as Oct4 is expressed in a variety of cancers, chemotherapy-induced Oct4 expression and the resultant drug resistance may also operate in different cancers. A growing body of evidence has shown multifunctional oncogenic transcription factors as potential targets for cancer therapy. Since cisplatin-based chemotherapy is common for cancer therapy, suppression of Oct4 expression by the pharmacological inhibitor ATRA or RNA interference-mediated silencing may provide a promising strategy for treating different cancers. This notion is clearly demonstrated in our results by the synergistic antitumor effects of combined treatment of cisplatin and ATRA on mice bearing Oct4-overexpressing human bladder tumor xenografts. Furthermore, our findings also reinforce the importance of precautions on preventing drug resistance via Oct4 when neoadjuvant chemotherapy is considered. Although we show that Oct4 induces acquired resistance of cancer cells to chemotherapeutic agents, the exact mechanisms of how anticancer drugs enhance Oct4 expression to confer drug resistance remain to be further investigated.

## MATERIALS AND METHODS

### Clinical specimens

This study analyzed 122 specimens of 110 consecutive patients with high-grade, stages T1-2 urothelial carcinoma of urinary bladder treated with TURBT between 2006 and 2010 at National Cheng Kung University (NCKU) Hospital. After TURBT, these patients received intravesical chemotherapy with epirubicin. None of the patients received neoadjuvant chemotherapy or preoperative radiotherapy. There was no metastatic disease at the time of surgery. This study was approved by Institutional Review Board of NCKU Hospital, and informed consent was obtained from each patient. Tumor recurrence was determined from hospital records.

### Cell culture and mice

Human bladder cancer cell lines (TCCSUP, J82, TSGH-8301, and T24) and human immortalized uroepithelial cell line (SV-HUC-1) have been previously described [44]. Cells were cultured in complete medium consisting of Dulbecco's modified Eagle's medium (DMEM) supplemented with 10% cosmic calf serum (Hyclone), 2 mmol/L L-glutamine, and 50 μg/mL gentamicin. Stable Oct4-overexpressing or Oct4-knockdown cells were established using lentiviral vectors and puromycin selection as described previously [54, 55]. Cisplatin-resistant TCCSUP cells were obtained by culturing cells in complete medium containing cisplatin (1 μg/ml). Male 6-8-week-old NOD/SCID mice were obtained from the Laboratory Animal Center of NCKU. All animal experiments were conducted following the guidelines approved by the Laboratory Animal Care and Use Committee of NCKU.

### Plasmids and lentiviral vectors

The lentiviral vector encoding human Oct4 was purchased from Addgene (pSin-EF2-Oct4-Pur, Addgene plasmid 16579). To construct the control lentiviral vector pSin-null, pSin-EF2-Oct4-Pur was digested with *Spe*I and *Bam*HI to excise the Oct4 cDNA, and the resulting large fragment was filled-in with T4 DNA polymerase (Takara) and subsequently self-ligated by T4 DNA ligase (Invitrogen). For knockdown experiments, pLKO.1-puro-based lentiviral vectors, including stem-loop cassettes encoding shRNA for human Oct4 (TRCN0000004882 and TRCN0000004883, designated shOct4 TRCN4882 and shOct4 TRCN4883) and luciferase (TRCN 0000072246, designated shLuc), were obtained from the National RNAi Core Facility, Academia Sinica, Taiwan. Lentiviruses were produced and titrated as previously described [55].

### Quantitative real-time RT-PCR analysis

Total RNA (2 μg) was reverse-transcribed into cDNA using the Verso™ cDNA synthesis kit (Thermo Fisher Scientific). Quantitative real-time RT-PCR was performed using a SmartCycler System (Cephid). Each reaction contained 50 ng cDNA, SYBR Premix Ex Taq (TaKaRa), and 5 μmol of each forward and reverse primer. The following primers were used: human Oct4, 5′-GTCCGAGTGTGGTTCTGTA-3′ (forward) and 5′-CTCAGTTTGAATGCATGGGA-3′ (reverse); human glyceraldehyde-3-phosphate dehydrogenase (GAPDH), 5′-ACTTCAACAGCGACACCCACT-3′ (forward) and 5′-GCCAAATTCGTTGTCATACCAG-3′ (reverse). Normalization was performed using GAPDH as the internal control, and relative gene expression was calculated using the comparative 2(-^ΔΔ^Ct) method [56].

### Immunohistochemistry, immunoblotting, and immunofluorescence

For immunohistochemistry, specificity of the anti-Oct4 antibody used in this study has been validated in our previous papers showing immunoreactivity with bladder cancer cells and ES cells, but not with normal cells [14, 44]. Immunohistochemistry was performed on serial formalin-fixed, paraffin-embedded sections of human and mouse bladder tissue after antigen retrieval with proteinase K. After blocking with 5% BSA, tissue sections were incubated with mouse anti-Oct4 monoclonal antibody (Santa Cruz, sc-5279) at 4°C overnight, followed by incubation with horseradish peroxidase (HRP)-conjugated goat anti-mouse IgG (H+L) (Jackson Laboratory, 115-035-003) at room temperature for 2 h. The reactivity was visualized with aminoethyl carbazole (red color, Invitrogen) and counterstained with hematoxylin. Immunoblot analysis was performed to detect Oct4, RARα, and β-actin (as the loading control) using rabbit anti-Oct4 antibody (Cell Signaling, #2750), goat anti-RARα (Abcam, ab28767), and anti-β-actin-peroxidase antibody (Sigma-Aldrich, A3854), respectively, as previously described [57]. For immunofluorescence staining, cancer cells were fixed in 4% paraformaldehyde for 15 min, treated with 0.1% Triton X-100 in PBS for 5 min at room temperature, and then blocked with 1% bovine serum albumin. Sections of formalin-fixed, paraffin-embedded human and mouse bladder tissue were also subjected to immunofluorescence staining, as previously described [58]. Cells and tumor tissue sections were then incubated with anti-CD44-PE antibody (Miltenyi Biotec, 130-098-108) and mouse anti-Oct4 monoclonal (Santa Cruz, sc-5279) at 4°C overnight. After being washed with PBS, they were sequentially incubated with Alexa Fluor488-goat anti-mouse IgG (H+L) (Life Technologies, A-11001) for 2 h at room temperature. The nucleus was counterstained with DAPI (50 μg/ml).

### Drug sensitivity assay and drug combination analysis

Various bladder cancer cells (2 × 10^3^) that were cultured in 96-well plates in complete medium overnight were refed with fresh medium containing various concentrations of chemotherapeutic drugs and cultured for 72 h. A colorimetric WST-8 assay (Dojindo Laboratories) was used to determine cell viability. The IC_50_ values were determined as the drug concentration at 50% inhibition of cell growth.

To analyze the effect of the drug combination, TCCSUP cells (4 × 10^3^) cultured in 96-well plates were treated with cisplatin ranging from 0 to 10 μg/ml plus ATRA (0.1 μM) or the solvent for 72 h. The cytotoxic effect was assessed by the WST-8 assay. The CDI was used to analyze drug combinations as previously described [22–24]. The CDI was calculated by the following formula: CDI = AB/(A × B). AB is the survival rate of the two-drug combination group relative to the control group, and A or B is the survival rate of the single drug group relative to the control group. The CDI values of < 1, = 1, and > 1 indicate that the drugs are synergistic, additive, and antagonistic, respectively.

### Animal experiments

NOD/SCID mice were inoculated subcutaneously with parental or cisplatin-resistant TCCSUP cells (1 × 10^7^) at day 0, followed by intraperitoneal injection of cisplatin (4 mg/kg) at days 5, 7, 9, 11, and 13. In addition, Oct4-overexpressing and control TCCSUP cells were employed for *in vivo* experiments. TCCSUP cells (2 × 10^6^) that had been transduced with lentiviral vectors encoding Oct4 or with control vectors were inoculated subcutaneously into the right flank of NOD/SCID mice at day 0. Subsequently, the mice were treated intraperitoneally with cisplatin (4 mg/kg) or saline when their tumor volumes reached 100 mm^3^. In other sets of the experiment, TCCSUP tumor-bearing NOD/SCID mice were treated intraperitoneally with cisplatin (4 mg/kg), ATRA (10 mg/kg), saline, or the vehicle (solvent) alone. Furthermore, the tumor-bearing mice were treated with cisplatin, cisplatin plus ATRA, cisplatin plus solvent, or saline. All of the mice were monitored for tumor growth. Tumor volumes were calculated as: length × width^2^ × 0.45.

### Statistical analysis

Recurrence-free curves were calculated by the Kaplan and Meier method and compared by the log-rank test. Differences in the expression levels of Oct4 between primary and recurrent tumor tissue were evaluated with the Mann-Whitney U test. Differences in tumor volume and cell viability between groups were compared by two-way analysis of variance (ANOVA) with repeated measures. The remaining data were analyzed by one-way ANOVA with Bonferroni post hoc test. The differences were considered significant if *P* values were < 0.05.

## SUPPLEMENTARY MATERIALS FIGURE


