# Supplementary material for: Chemotherapeutics-induced Oct4 expression contributes to drug resistance and tumor recurrence in bladder cancer
Source: Oncotarget. 2016 May 26;8(19):30844–58. doi: 10.18632/oncotarget.9602 (PMC5458172; doi:10.18632/oncotarget.9602)
Supplement: Supplementary file 1 [file oncotarget-08-30844-s001.pdf]

SUPPLEMENTARY FIGURE

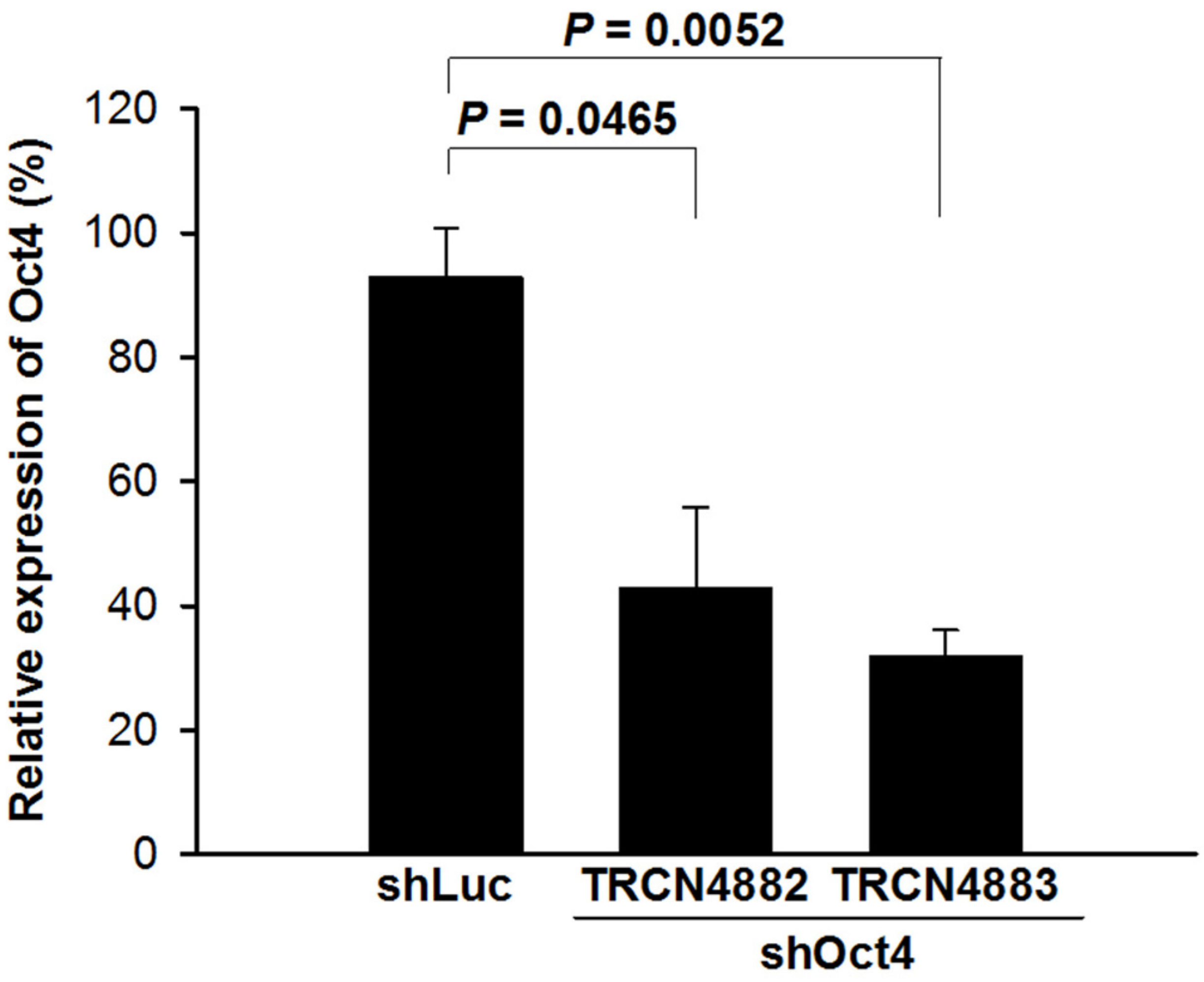

Supplementary Figure S1: Detection of Oct4 mRNA levels in Oct4 knockdown bladder cancer cells. TCCSUP cells transduced with lentiviruses expressing Oct4 shRNA (shOct4) or luciferase shRNA (shLuc) were assessed for Oct4 mRNA levels by quantitative real-time RT-PCR analysis. Values shown are the mean ± SEM (n = 3-4). Results are representatives of two independent experiments.
